# The association between dyslipidaemia in the first trimester and adverse pregnancy outcomes in pregnant women with subclinical hypothyroidism: a cohort study

**DOI:** 10.1186/s12944-023-01998-7

**Published:** 2024-01-11

**Authors:** Xueran Wang, Enjie Zhang, Zongyuan Tian, Rong Zhao, Kaikun Huang, Shen Gao, Shaofei Su, Shuanghua Xie, Jianhui Liu, Yingyi Luan, Yue Zhang, Zheng Zhang, Yousheng Yan, Wentao Yue, Chenghong Yin, Ruixia Liu

**Affiliations:** 1 Department of Central Laboratory, Beijing Obstetrics and Gynecology Hospital, Capital Medical University, Beijing Maternal and Child Health Care Hospital, Beijing, 100026 China; 2 Department of Obstetrics, Beijing Obstetrics and Gynecology Hospital, Capital Medical University, Beijing Maternal and Child Health Care Hospital, Beijing, 100026 China; 3 Department of Prenatal Diagnosis Center, Beijing Obstetrics and Gynecology Hospital, Capital Medical University, Beijing Maternal and Child Health Care Hospital, Beijing, 100026 China

**Keywords:** Dyslipidemia, Pregnancy, Subclinical hypothyroidism, Pregnancy outcomes

## Abstract

**Background:**

Subclinical hypothyroidism (SCH) is linked to dyslipidaemia and adverse pregnancy outcomes. However, the impact of dyslipidaemia on the outcome of pregnancy in SCH is unclear.

**Methods:**

We enrolled 36,256 pregnant women and evaluated their pregnancy outcomes. The following data was gathered during the first trimester (≤ 13^+ 6^ weeks of gestation): total cholesterol (TC), low-density lipoprotein (LDL-C), triglyceride (TG), high-density lipoprotein (HDL-C), free thyroxine (FT4) and thyroid-stimulating hormone (TSH) concentrations. The reference ranges for lipids were estimated to range from the 5th to the 95th percentile. Logistic regression assessed the relationships between dyslipidaemia and adverse pregnancy outcomes, including abortion, preeclampsia/eclampsia, low birth weight, foetal growth restriction, premature rupture of foetal membranes, gestational hypertension, preterm birth, macrosomia and gestational diabetes mellitus (GDM). Additionally, the best thresholds for predicting adverse pregnancy outcomes based on TSH, FT4, and lipid levels were determined using receiver operating characteristic curves.

**Results:**

In the first trimester, LDL-C > 3.24 mmol/L, TG > 1.92 mmol/L, HDL-C < 1.06 mmol/L, and TC > 5.39 mmol/L were used to define dyslipidaemia. In this cohort, 952 (3.56%) patients were diagnosed with SCH, and those who had dyslipidaemia in the first trimester had higher incidences of gestational hypertension (6.59% vs. 3.25%), preeclampsia/eclampsia (7.14% vs. 3.12%), GDM (22.53% vs. 13.77%), and low birth weight (4.95% vs. 2.08%) than did those without dyslipidaemia. However, after adjusting for prepregnancy body mass index (pre-BMI), dyslipidaemia was no longer related to these risks. Furthermore, elevated TG dyslipidaemia in SCH patients was connected to an enhanced potential of gestational hypertension (odds ratio [OR]: 2.687, 95% confidence interval [CI]: 1.074 ~ 6.722), and elevated LDL-C dyslipidaemia correlated with increased preeclampsia/eclampsia risk (OR: 3.172, 95% CI: 1.204 ~ 8.355) after accounting for age, smoking status, alcohol use, pre-BMI, and levothyroxine use. Additionally, the combination of TC, TG, LDL-C, pre-BMI, and TSH exhibited enhanced predictive capabilities for gestational hypertension, preeclampsia/eclampsia, and GDM. Values of 0.767, 0.704, and 0.706 were obtained from the area under the curve.

**Conclusions:**

Among pregnant women with SCH, dyslipidaemia in early pregnancy was related to elevated risks of adverse pregnancy consequences. The combined consideration of age, pre-BMI, TSH, and lipid levels in the first trimester could be beneficial for monitoring patients and implementing interventions to reduce adverse pregnancy outcomes.

## Background

During pregnancy, lipids are physiologically elevated to accommodate the increased needs of both the mother and foetus. However, excessive lipid levels can lead to lipotoxicity, potentially impacting placental development, inflammation, and metabolism, thereby contributing to adverse perinatal outcomes and potential health issues for the offspring [1]. Elevated maternal triglyceride (TG) and apolipoprotein levels are related to an increased likelihood of macrosomia and preeclampsia [2, 3]. A meta-analysis of 31,402 pregnancies reveals that increased maternal TG also decreased high-density lipoprotein cholesterol (HDL-C) values during pregnancy particularly in overweight or obese women are connected to an increased probability of large for gestational age (LGA) infants and macrosomia and a decreased likelihood of small-for-gestational-age (SGA) infants [4]. Additionally, increased low-density lipoprotein cholesterol (LDL-C) concentrations are related to a higher likelihood of preterm birth [5]. Notably, maternal hypercholesterolemia during pregnancy leads to alterations in the foetal aorta, potentially predisposing the offspring to atherosclerosis later in life [6]. However, the underlying cause of dyslipidaemia during pregnancy remains unclear. Moreover, there are currently no established recommendations for lipid reference ranges during pregnancy, and the lack of safety data has hindered the development of effective medications for controlling dyslipidaemia during pregnancy [7].

Subclinical hypothyroidism (SCH) is the most commonly observed thyroid disorder during pregnancy; SCH is defined by normal free thyroxine (FT4) values and raised thyroid stimulating hormone (TSH) concentrations [8]. Elevated TSH levels are significantly correlated with an unfavourable lipid profile [9] and could serve as a risk factor for dyslipidaemia during pregnancy. Furthermore, several convincing meta-analyses have demonstrated a connection between SCH during pregnancy and an increased incidence of SGA infants, lower birthweights, preeclampsia, and preterm birth [10–12]. Two smaller studies have identified differences in lipid molecules and metabolites between pregnant women with SCH or hypothyroidism and euthyroid individuals, suggesting a potential link between lipid profiles and the pathogenesis of thyroid disorders during pregnancy, in addition to unfavourable pregnancy outcomes [13, 14].

There are complex interactions between thyroid-related hormones and lipids during pregnancy. However, it is unclear how dyslipidaemia affects outcomes in patients with SCH during pregnancy. For this reason, the study was designed to discuss the connection between dyslipidaemia in early pregnancy and unfavourable pregnancy outcomes in pregnant women with SCH.

## Methods

### Participants

The cohort study enrolled pregnant women who established archives at the Beijing Obstetrics and Gynecology Hospital, Capital Medical University, from February 2018 to December 2020 and who had taken part in the China Birth Cohort Study (CBCS) [15]. All participants were asked to complete questionnaires, underwent clinical laboratory measurements, and were followed up with to assess pregnancy outcomes. To establish reference ranges for lipids in the first trimester, pregnant women who satisfied the following criteria were excluded: (1) dropped out after enrolment; (2) pregnancies with non-singletons; (3) were outside the range of 18 to 50 years old; (4) had incomplete lipid data in the first trimester; (5) had preexisting conditions before pregnancy (e.g., severe cardiac, hepatic, or renal disorders; hypertension; diabetes; thyroid disorders; malignant tumours or postoperative tumours; infectious diseases; or hyperlipidaemia); (6) had used of lipid-affecting medications prior to pregnancy; (7) had pregnancies achieved through assisted reproductive technologies; and (8) were lost to follow-up regarding pregnancy outcomes. Furthermore, during the screening process for pregnant women with SCH, who matched at least one of these conditions were excluded: had used medications affecting thyroid function before pregnancy (e.g., levothyroxine (L-T4), prednisone, budesonide, dexamethasone, propylthiouracil, labetalol, methylprednisolone); had TSH levels in the normal ranges of reference; had TSH levels lower than normal reference ranges; or had FT4 concentrations outside of the normal range. This study received approval from the Ethics Committee of Beijing Obstetrics and Gynecology Hospital, Capital Medical University on February 2nd, 2018 (reference number: 2018-KY-003-02). All participants provided informed consent.

### Data acquisition

Baseline data were collected using the questionnaires from the CBCS and the medical records at the hospital. Age was calculated using the birthdate and enrolment date. Prepregnancy body mass index (pre-BMI) was obtained by dividing prepregnancy weight in kilograms by height in meters squared. Smoking status and alcohol consumption during pregnancy were categorized as “yes”. Education levels were classified into three groups: high school and below, undergraduate, and graduate. Additionally, prepregnancy health conditions and medication usage were verified through a combination of questionnaires and medical records. The use of L-T4 before and during pregnancy was confirmed through questionnaires, hospital records, and phone follow-ups.

During the first trimester (≤ 13^+ 6^ weeks gestation), serum samples were collected from those enrolled in the CBCS study and archived at Beijing Obstetrics and Gynecology Hospital, Capital Medical University. The samples were maintained at 4 °C for less than 24 h, and the test was completed on the day of sample collection. Then, if samples need extended storage, they were kept at -20 °C for 48 h or -80 °C for more than three months. TSH and FT4 serum levels were collected via electrochemiluminescence immunoassays (Siemens Healthcare Diagnostics, ADVIA Centaur XP, Tarrytown, NY/USA). A fully automated Integrated System Chemistry/Immunology Analyser (ARCHITECT ci16200, Abbott Park, IL, USA) was used to quantify lipid levels, comprising LDL-C, HDL-C, TG, and total cholesterol (TC) using the appropriate assay kits (reagent cat. No. H05119R02 for TC, G07893R02 for TG, G05251R03 for HDL-C, and G69452R13 for LDL-C, Abbott Park, IL, USA). Initial blood lipid and thyroid function test results in early pregnancy were utilized as key indicators in this investigation.

### Definitions

During early pregnancy, SCH was diagnosed by normal concentrations of FT4 and TSH concentrations above 4.0 mIU/L, which is in accordance with the 2017 American Thyroid Association Guidelines [16]. As per our hospital’s standards, the normal reference range for FT4 was established to be 11.8–18.4 pmol/L.

Reference ranges for LDL-C, TG, HDL-C, and TC in early pregnancy were established in this study. Dyslipidaemia was identified by elevated TC, elevated TG, elevated LDL, and reduced HDL according to the reference range in this study. Adverse pregnancy outcomes included abortion (a pregnancy terminated before 28 weeks and with foetal weight less than 1000 g, including artificial abortion and spontaneous abortion), gestational hypertension (diastolic blood pressure ≥ 90 mmHg and/or systolic blood pressure ≥ 140 mmHg that appears after the 20th week of pregnancy and becomes normal within 12 weeks of postpartum, and accompanied by a negative urine protein test), gestational diabetes mellitus (GDM, diagnosed if any following ranges is exceeded: 2-hour post glucose load ≤ 8.5 mmol/L, 1-hour post glucose load ≤ 10.0 mmol/L, fasting blood glucose ≤ 5.1 mmol/L), preeclampsia/eclampsia (meeting criteria for gestational hypertension coupled with significant proteinuria or convulsions arising from preeclampsia without other explanatory causes), premature rupture of foetal membranes (PROM, identified as preterm membrane rupture before 37 weeks of gestation), foetal growth restriction (FGR, characterized by foetuses whose estimated foetal weight falls below the 10th gestational age percentile) [17], low birth weight (regardless of gestational age, birth weight below 2,500 g), macrosomia (neonatal birthweight is equal to or more than 4,000 g, irrespective of gestational age), preterm birth (described as a premature birth before 37 weeks of pregnancy), and birth defects (inclusive of structural deformities and functional abnormalities). The analyses also included modes of delivery, comprising vaginal delivery (encompassing both natural childbirth and operative vaginal birth) and caesarean section.

### Statistical analyses

Continuous variables with a normal distribution were displayed in both average and standard deviations; nonnormally distributed values were then in the form of medians and interquartile ranges. For categorical variables, standard deviation and median were used. Subsequently, reference ranges were then established for the first trimester of pregnancy using the 5th to 95th percentiles of lipid measurements. Differences between groups were investigated using Mann-Whitney U and chi-square tests. The relationships between dyslipidaemia and pregnancy consequences were investigated by logistic regression analysis. The adjusted model included age, pre-BMI, smoking status, alcohol use, and L-T4 use during pregnancy as sequential adjustment factors. Receiver operating characteristic (ROC) curve determined the threshold levels for TSH, FT4, lipid levels, age, and pre-BMI for predicting adverse outcomes in pregnant women with SCH. Prediction accuracy was analysed by area under the curve (AUC). Youden index was adopted to find the optimal trade-off between sensitivity and specificity. Analysis of data was carried out with SPSS 26.0 (SPSS, Inc., Chicago, IL, USA). *P* < 0.05 was seen as an indicator of statistical significance.

## Results

### First-trimester serum lipid reference range

Among the enrolled pregnant women, 26,776 were included in the analysis to establish reference values. Table 1 displays the average TC, HDL-C, TG, and LDL-C concentrations in the first trimester, and also the respective percentiles. As it is customary to use the 95th percentiles to define the normal reference range [18], the first-trimester reference ranges for serum LDL-C, TG, HDL-C, and TC were set between the 5th and 95th percentiles. Therefore, the following reference values for the first trimester: TC: 3.20 ~ 5.39 mmol/L; TG: 0.56 ~ 1.92 mmol/L; HDL-C: 1.06 ~ 2.01 mmol/L; and LDL-C: 1.37 ~ 3.24 mmol/L.

**Table 1:** Serum lipids of pregnant women in the first trimester (n = 26,776)

	TC (mmol/L)	TG (mmol/L)	LDL-C (mmol/L)	HDL-C (mmol/L)
Percentile				
2.5%	3.03	0.51	1.22	0.99
5%	3.20	0.56	1.37	1.06
10%	3.39	0.62	1.53	1.15
25%	3.74	0.76	1.82	1.30
50%	4.15	0.96	2.17	1.48
75%	4.62	1.25	2.56	1.68
90%	5.07	1.62	2.96	1.88
95%	5.39	1.92	3.24	2.01
97.5%	5.68	2.27	3.50	2.13

*Abbreviations: TC* total cholesterol, *TG* triglycerides, *HDL-C* high-density lipid cholesterol, *LDL-C* low-density lipid cholesterol

### Baseline information

**Fig. 1:**
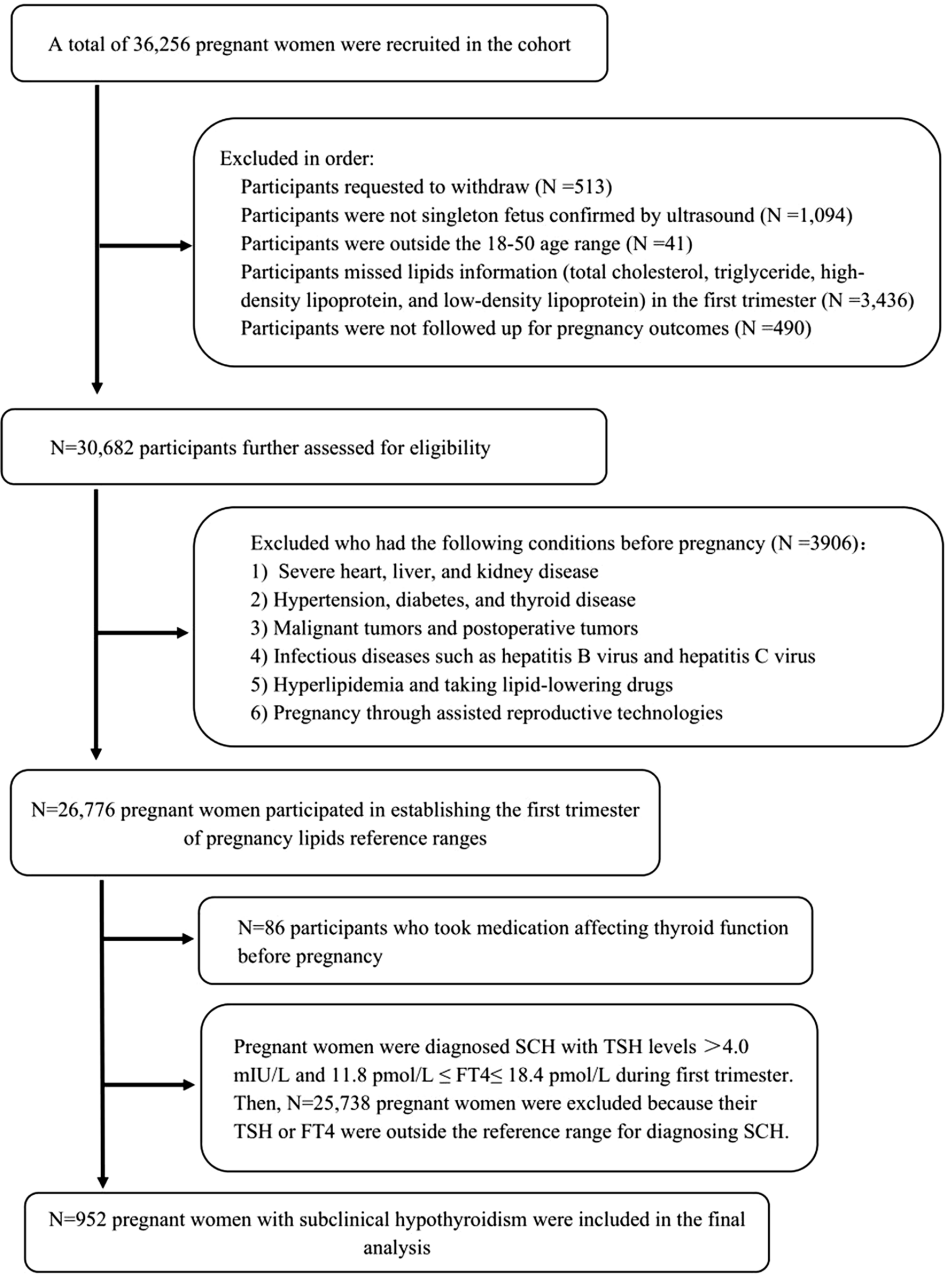
The flowchart of study participants. *Abbreviations: TSH* thyroid-stimulating hormone; *FT4* free thyroxine

In total, 952 pregnant women were identified with SCH. The flowchart is depicted in Fig. 1. Among these pregnant women with SCH, 770 (80.88%) presented without dyslipidaemia, while 182 (19.12%) presented with dyslipidaemia. Pregnant women with dyslipidaemia were characterized by advanced maternal age (32.00 years vs. 30.00 years) and had a greater pre-BMI (23.03 kg/m^2^ vs. 20.76 kg/m^2^) than did those without dyslipidaemia. Additionally, a greater proportion of women in the dyslipidaemia group were classified as older (age ≥ 35 years) or obese (pre-BMI ≥ 28 kg/m^2^). In the first trimester of pregnancy, among those with SCH, those with dyslipidaemia exhibited a greater incidence of gestational hypertension (6.59% vs. 3.25%), preeclampsia/eclampsia (7.14% vs. 3.12%), GDM (22.53% vs. 13.77%), and low birth weight (4.95% vs. 2.08%). No significant disparities in other pregnancy outcomes were evident between the dyslipidaemia and the nondyslipidaemia group (Table 2).

**Table 2:** Characteristics of the pregnant women with SCH in the first trimester

	Total (n = 952)	SCH without dyslipidemia (n = 770)	SCH with dyslipidemia (n = 182)	*P*-value
Maternal Age (year)	31.00(28.00, 33.00)	30.00(28.00, 33.00)	32.00(29.00, 34.25)	＜0.001^*^
< 35	777(81.62)	640(83.12)	137(75.27)	0.014*
≥ 35	175(18.38)	130(16.88)	45(24.73)	
Pre-pregnancy BMI (kg/m^2^)	21.09(19.49, 23.70)	20.76(19.22, 23.05)	23.03(20.56, 26.01)	＜0.001^*^
< 24	738(77.52)	630(81.82)	108(59.34)	0.000*
24–28	157(16.49)	110(14.29)	47(25.82)	
≥ 28	57(5.99)	30(3.90)	27(14.84)	
Smoking (Yes)	3(0.32)	1(0.14)	2(1.20)	0.092
Drinking (Yes)	42(4.41)	36(4.68)	6(3.30)	0.415
Education level				0.250
< College	69(7.25)	51(7.03)	18(10.78)	
Undergraduate/College	621(65.23)	507(69.93)	114(68.26)	
>Postgraduate/higher	202(21.22)	167(23.03)	35(20.96)	
TSH (mIU/L)	4.89(4.34, 5.75)	4.89(4.34, 5.74)	4.90(4.35, 5.77)	0.630
FT4 (pmol/L)	14.72(13.82, 15.81)	14.70(13.80, 15.74)	14.75(13.99, 16.07)	0.168
TC (mmol/L)	4.22(3.76, 4.70)	4.13(3.74, 4.54)	5.04(4.01, 5.55)	＜0.001^*^
TG (mmol/L)	0.99(0.78, 1.35)	0.92(0.73, 1.19)	1.67(1.21, 2.24)	＜0.001^*^
LDL-C (mmol/L)	2.19(1.84, 2.64)	2.12(1.81, 2.48)	2.87(2.12, 3.40)	＜0.001^*^
HDL-C (mmol/L)	1.49(1.28, 1.72)	1.52(1.33, 1.73)	1.33(1.05, 1.68)	＜0.001^*^
Vaginal delivery	570(59.87)	465(60.39)	105(57.69)	0.505
Cesarean Section	333(34.98)	265(34.42)	68(37.36)	0.454
Pregnancy loss	49(5.15)	40(5.19)	9(4.95)	0.891
Gestational hypertension	37(3.89)	25(3.25)	12(6.59)	0.036^*^
Preeclampsia/Eclampsia	37(3.89)	24(3.12)	13(7.14)	0.012^*^
GDM	147(15.44)	106(13.77)	41(22.53)	0.003^*^
PROM	279(29.31)	227(29.48)	52(28.57)	0.809
FGR	93(9.77)	72(9.35)	21(11.54)	0.372
Preterm birth	32(3.36)	23(2.99)	9(4.95)	0.188
Low birth weight	25(2.63)	16(2.08)	9(4.95)	0.030^*^
Macrosomia	59(6.20)	46(5.97)	13(7.14)	0.557
Birth defects	37(3.89)	31(4.03)	6(3.30)	0.647

*Abbreviations: SCH* subclinical hypothyroidism; *BMI* body mass index; *TSH* thyroid-stimulating hormone; *FT4* free thyroxine; *TC* total cholesterol; *TG* triglyceride; *LDL-C* low density lipoprotein cholesterol; *HDL-C* high density lipoprotein cholesterol; *GDM* gestational diabetes mellitus; *PROM*, premature rupture of fetal membranes; *FGR* fetal growth restriction

Data are median (interquartile range) or n (%)

^*^P-values < 0.05 was considered statistically significant

### Adverse pregnancy outcome risk in patients with dyslipidaemia

Compared to women with SCH who did not have dyslipidaemia, those with dyslipidaemia exhibited an increased risk of developing gestational hypertension (odds ratio [OR]: 2.104, 95% confidence interval [CI] [1.036 ~ 4.271], *P* = 0.040), preeclampsia/eclampsia (OR: 2.391, 95% CI: 1.193 ~ 4.792, *P* = 0.014), GDM (OR: 1.821, 95% CI: 1.216 ~ 2.727, *P* = 0.004), and low birth weight (OR: 2.452, 95% CI: 1.066 ~ 5.640, *P* = 0.035) in the crude model. However, after accounting for confounding factors, the association lacked statistical significance (Table 3).

**Table 3:** Odds ratios of adverse outcomes in SCH with dyslipidemia compared with those without dyslipidemia

Maternal and neonatal outcomes		Without dyslipidemia		Dyslipidemia			Dyslipidemia	
				Crude	*P*-values		Adjusted	*P*-values
Vaginal delivery		reference		0.894 (0.645–1.241)	0.504		1.238 (0.858–1.785)	0.253
Caesarean Section		reference		1.137 (0.813–1.589)	0.454		0.851 (0.586–1.236)	0.396
Pregnancy loss		reference		0.949 (0.452–1.994)	0.891		0.783 (0.345–1.773)	0.557
Gestational hypertension		reference		2.104 (1.036–4.271)	0.040^*^		1.996 (0.913–4.364)	0.083
Preeclampsia/Eclampsia		reference		2.391 (1.193–4.792)	0.014^*^		1.557 (0.680–3.568)	0.295
GDM		reference		1.821 (1.216–2.727)	0.004^*^		1.427 (0.911–2.325)	0.121
PROM		reference		0.957 (0.670–1.367)	0.809		1.009 (0.684–1.490)	0.964
FGR		reference		1.264 (0.755–2.117)	0.372		1.293 (0.733–2.282)	0.375
Preterm birth		reference		1.690 (0.768–3.716)	0.192		1.308 (0.550–3.114)	0.543
Low birth weight		reference		2.452 (1.066–5.640)	0.035^*^		1.923 (0.744–4.976)	0.177
Macrosomia		reference		1.211 (0.640–2.291)	0.557		0.632 (0.269–1.483)	0.291
Birth defects		reference		0.813 (0.334–1.978)	0.648		0.837 (0.333–2.107)	0.706

*Abbreviations: SCH* subclinical hypothyroidism; *BMI* body mass index; *GDM* gestational diabetes mellitus; *PROM*, premature rupture of fetal membranes; *FGR* fetal growth restriction; *L-T4* levothyroxine

Crude: No adjustment. Adjusted: L-T4 use, age, alcohol use, smoking, and pre-BMI.

^*^P-values < 0.05 was considered statistically significant

As shown in Table 4, after adjusting for L-T4 use and age, dyslipidaemia still raised the risk of the above four undesirable pregnancy outcomes. After including smoking status and alcohol use as adjustment variables, dyslipidaemia in the first trimester was only linked to an enhanced risk of gestational hypertension (OR: 2.810, 95% CI: 1.321 ~ 5.975, *P* = 0.007) and GDM (OR: 1.702, 95% CI: 1.101 ~ 2.632, *P* = 0.017) in women with SCH. Moreover, after adjusting for pre-BMI, the correlation did not reach statistical significance.

**Table 4:** Multivariable regression results for several adverse pregnancy outcomes

Maternal and neonatal outcomes	Without dyslipidemia		Dyslipidemia	
			OR (95%CI)	*P*-values
Gestational hypertension				
Model 1	reference		2.171 (1.066–4.421)	0.033^*^
Model 2	reference		2.092 (1.017–4.301)	0.045^*^
Model 3	reference		2.810 (1.321–5.975)	0.007^*^
Model 4	reference		1.996 (0.913–4.364)	0.083
Preeclampsia/Eclampsia				
Model 1	reference		2.400 (1.197–4.813)	0.014^*^
Model 2	reference		2.366 (1.171–4.777)	0.016^*^
Model 3	reference		1.878 (0.835–4.225)	0.128
Model 4	reference		1.557 (0.680–3.568)	0.295
GDM				
Model 1	reference		1.870 (1.245–2.809)	0.003^*^
Model 2	reference		1.609 (1.059–2.446)	0.026^*^
Model 3	reference		1.702 (1.101–2.632)	0.017^*^
Model 4	reference		1.427 (0.911–2.325)	0.121
Low birth weight				
Model 1	reference		2.462 (1.070–5.668)	0.034^*^
Model 2	reference		2.422 (1.043–5.624)	0.040^*^
Model 3	reference		2.114 (0.838–5.332)	0.113
Model 4	reference		1.923 (0.744–4.976)	0.177

*Abbreviations: GDM* gestational diabetes mellitus; *BMI* body mass index; *L-T4* levothyroxine

Model 1: Adjusted for L-T4 use

Model 2: Adjusted for the same variables as Model 1 as well as age

Model 3: Adjusted for the same variables as Model 2 as well as d alcohol use and smoking

Model 4: Adjusted for the same variables as Model 3 as well as pre-BMI.

^*^P-values < 0.05 was considered statistically significant

### Associations between different types of dyslipidaemia and pregnancy outcomes

According to the crude model, among pregnant women with SCH, elevated TC dyslipidaemia was correlated with increased preeclampsia/eclampsia risk, while elevated TG dyslipidaemia was linked to increased risks of gestational hypertension, GDM, and low birth weight. Elevated LDL-C dyslipidaemia was correlated with an elevated incidence of GDM and preeclampsia/eclampsia, and reduced HDL-C dyslipidaemia was associated with low birth weight. However, after adjusted for age, L-T4 use, smoking status, and alcohol use, there were still the significant associations between elevated TG dyslipidaemia and an increased gestational hypertension risk (OR: 4.064, 95% CI: 1.691 ~ 9.763, *P* = 0.002) and GDM (95% CI: 1.071 ~ 3.341, *P* = 0.028), as well as between elevated LDL-C dyslipidaemia and increased preeclampsia/eclampsia risk (OR: 3.870, 95% CI: 1.502 ~ 9.966, *P* = 0.005) and between reduced HDL-C dyslipidaemia and increased low birth weight incidence (OR: 3.581, 95% CI: 1.012 ~ 12.666, *P* = 0.048). Furthermore, after incorporating pre-BMI as an adjustment variable, only the associations between elevated TG dyslipidaemia and the risk of gestational hypertension (OR: 2.687, 95% CI: 1.074 ~ 6.722, *P* = 0.035) and between elevated LDL-C dyslipidaemia and preeclampsia/eclampsia (OR: 3.172, 95% CI: 1.204 ~ 8.355, *P* = 0.019) continued to be significant (Table 5).

**Table 5:** Association of lipid levels with pregnancy outcomes in pregnant women with SCH

Maternal and neonatal outcomes	Elevated TC			Elevated TG			Elevated LDL-C			Reduced HDL-C	
	OR (95%CI)	*P*-values		OR (95%CI)	*P*-values		OR (95%CI)	*P*-values		OR (95%CI)	*P*-values
Vaginal delivery											
Crude	0.803 (0.494–1.307)	0.378		0.595 (0.377–0.939)	0.026^*^		0.724 (0.430–1.218)	0.224		1.123 (0.617–2.046)	0.703
Model 1	0.933 (0.567–1.535)	0.784		0.715 (0.446–1.145)	0.162		0.801 (0.471–1.363)	0.413		1.154 (0.626–2.129)	0.647
Model 2	0.943 (0.559–1.591)	0.826		0.678 (0.413–1.112)	0.124		0.849 (0.490–1.470)	0.559		1.293 (0.657–2.548)	0.457
Model 3	1.035 (0.609–1.759)	0.899		0.819 (0.492–1.364)	0.443		1.006 (0.574–1.762)	0.984		1.490 (0.750–2.960)	0.255
Cesarean Section											
Crude	1.230 (0.749–2.019)	0.414		1.546 (0.976–2.449)	0.063		1.314 (0.775–2.230)	0.311		0.926 (0.500-1.713)	0.806
Model 1	1.101 (0.665–1.824)	0.708		1.347 (0.842–2.155)	0.213		1.220 (0.715–2.084)	0.466		0.927 (0.498–1.725)	0.810
Model 2	1.108 (0.652–1.884)	0.705		1.408 (0.861–2.301)	0.172		1.159 (0.665–2.021)	0.603		0.813 (0.404–1.635)	0.562
Model 3	0.997 (0.580–1.714)	0.992		1.139 (0.685–1.893)	0.616		0.962 (0.544-1.700)	0.893		0.685 (0.336–1.398)	0.299
Pregnancy loss											
Crude	1.109 (0.387–3.177)	0.847		1.540 (0.635–3.737)	0.339		1.319 (0.458–3.797)	0.607		0.793 (0.187–3.366)	0.753
Model 1	1.075 (0.344–3.358)	0.900		1.282 (0.479–3.432)	0.620		1.277 (0.401–4.064)	0.679		0.702 (0.151–3.252)	0.651
Model 2	0.948 (0.300-2.994)	0.928		1.260 (0.458–3.467)	0.655		1.156 (0.358–3.739)	0.808		0.781 (0.166–3.683)	0.755
Model 3	0.959 (0.303–3.039)	0.944		1.325 (0.467–3.759)	0.597		1.192 (0.363–3.911)	0.772		0.781 (0.166–3.685)	0.755
Gestational hypertension											
Crude	1.534 (0.528–4.460)	0.432		3.182 (1.404–7.213)	0.006^*^		0.829 (0.195–3.531)	0.800		1.706 (0.505–5.766)	0.390
Model 1	1.460 (0.493–4.324)	0.495		3.146 (1.350–7.328)	0.008^*^		0.797 (0.186–3.424)	0.760		1.754 (0.516–5.968)	0.368
Model 2	1.851 (0.613–5.590)	0.275		4.064 (1.691–9.763)	0.002^*^		0.947 (0.218–4.108)	0.942		2.420 (0.696–8.420)	0.165
Model 3	1.426 (0.457–4.454)	0.541		2.687 (1.074–6.722)	0.035^*^		0.570 (0.126–2.576)	0.465		1.676 (0.468-6.000)	0.428
Preeclampsia/Eclampsia											
Crude	3.103 (1.312–7.339)	0.010^*^		1.725 (0.653–4.556)	0.271		3.720 (1.563–8.857)	0.003^*^		2.399 (0.814–7.072)	0.113
Model 1	3.049 (1.275–7.291)	0.012^*^		1.665 (0.620–4.470)	0.312		3.664 (1.531–8.771)	0.004^*^		2.407 (0.816-7.100)	0.111
Model 2	1.948 (0.649–5.854)	0.235		1.136 (0.329–3.917)	0.840		3.870 (1.502–9.966)	0.005^*^		2.531 (0.731–8.765)	0.143
Model 3	1.706 (0.561–5.188)	0.346		0.855 (0.241–3.027)	0.808		3.172 (1.204–8.355)	0.019^*^		2.086 (0.595–7.320)	0.251
GDM											
Crude	1.666 (0.926–2.995)	0.088		2.225 (1.316–3.762)	0.003^*^		1.875 (1.017–3.456)	0.044^*^		1.101 (0.504–2.402)	0.810
Model 1	1.341 (0.726–2.477)	0.349		1.760 (1.016–3.047)	0.044^*^		1.666 (0.882–3.146)	0.116		1.127 (0.506–2.510)	0.769
Model 2	1.475 (0.783–2.780)	0.229		1.891 (1.071–3.341)	0.028^*^		1.789 (0.936–3.419)	0.078		1.018 (0.409–2.532)	0.970
Model 3	1.289 (0.675–2.464)	0.442		1.492 (0.830–2.681)	0.181		1.447 (0.746–2.806)	0.275		0.808 (0.320–2.043)	0.653
PROM											
Crude	0.807 (0.464–1.404)	0.447		1.084 (0.662–1.776)	0.748		0.774 (0.425–1.411)	0.404		1.219 (0.658–2.259)	0.530
Model 1	0.937 (0.522–1.681)	0.826		1.140 (0.690–1.884)	0.609		0.794 (0.434–1.453)	0.454		1.242 (0.668–2.309)	0.494
Model 2	0.880 (0.491–1.576)	0.667		1.250 (0.740–2.111)	0.404		0.816 (0.435–1.531)	0.527		1.253 (0.631–2.490)	0.520
Model 3	0.902 (0.500-1.625)	0.731		1.162 (0.679–1.988)	0.584		0.758 (0.401–1.436)	0.396		1.188 (0.594–2.376)	0.627
FGR											
Crude	1.189 (0.551–2.565)	0.659		0.863 (0.386–1.934)	0.721		1.008 (0.422–2.409)	0.985		1.927 (0.873–4.251)	0.104
Model 1	1.241 (0.571–2.697)	0.585		0.903 (0.399–2.043)	0.807		1.038 (0.433–2.490)	0.933		1.955 (0.885–4.321)	0.098
Model 2	1.398 (0.628–3.063)	0.403		0.668 (0.259–1.722)	0.404		1.106 (0.459–2.665)	0.823		1.335 (0.508–3.508)	0.558
Model 3	1.471 (0.668–3.239)	0.338		0.734 (0.281–1.918)	0.527		1.212 (0.497–2.958)	0.672		1.455 (0.548–3.861)	0.451
Preterm birth											
Crude	1.296 (0.385–4.364)	0.675		2.057 (0.770–5.494)	0.150		1.537 (0.455–5.197)	0.489		2.844 (0.956–8.464)	0.060
Model 1	1.242 (0.365–4.225)	0.729		1.982 (0.728–5.398)	0.181		1.495 (0.440–5.079)	0.519		2.867 (0.960–8.543)	0.059
Model 2	1.463 (0.425–5.035)	0.547		1.699 (0.562–5.133)	0.347		1.685 (0.491–5.778)	0.407		2.647 (0.764–9.176)	0.125
Model 3	1.226 (0.350–4.290)	0.750		1.189 (0.377–3.751)	0.768		1.261 (0.358–4.438)	0.718		2.136 (0.608–7.511)	0.237
Low birth weight											
Crude	1.723 (0.503–5.901)	0.387		2.799 (1.022–7.668)	0.045^*^		2.043 (0.594–7.026)	0.257		5.140 (1.841–14.348)	0.002^*^
Model 1	1.666 (0.481–5.771)	0.421		2.744 (0.979–7.693)	0.055		1.995 (0.557-6.900)	0.275		5.165 (1.848–14.434)	0.002^*^
Model 2	2.092 (0.593–7.376)	0.251		1.770 (0.498–6.290)	0.377		2.375 (0.677–8.326)	0.177		3.581 (1.012–12.666)	0.048^*^
Model 3	1.942 (0.546–6.911)	0.305		1.517 (0.411–5.597)	0.531		2.101 (0.584–7.556)	0.256		3.249 (0.906–11.653)	0.071
Macrosomia											
Crude	1.160 (0.449-3.000)	0.759		2.052 (0.970–4.343)	0.060		0.771 (0.234–2.539)	0.669		0.646 (0.153–2.729)	0.552
Model 1	1.251 (0.480–3.260)	0.646		2.334 (1.084–5.027)	0.030^*^		0.814 (0.246–2.689)	0.735		0.646 (0.153–2.733)	0.553
Model 2	0.594 (0.140–2.524)	0.480		1.761 (0.707–4.387)	0.224		0.655 (0.154–2.783)	0.567		0.440 (0.059–3.279)	0.423
Model 3	0.526 (0.122–2.261)	0.388		1.404 (0.540–3.651)	0.486		0.527 (0.122–2.289)	0.393		0.380 (0.051–2.858)	0.348
Birth defects											
Crude	1.099 (0.329–3.671)	0.878		0.605 (0.143–2.561)	0.495		1.304 (0.389–4.372)	0.667		1.080 (0.252–4.627)	0.918
Model 1	1.085 (0.322–3.656)	0.896		0.587 (0.137–2.517)	0.474		1.294 (0.384–4.360)	0.677		1.087 (0.254–4.664)	0.910
Model 2	1.213 (0.356–4.130)	0.757		0.638 (1.147–2.758)	0.547		1.404 (0.414–4.764)	0.586		1.365 (0.315–5.926)	0.678
Model 3	0.823 (0.336–3.944)	0.823		0.563 (0.128–2.481)	0.448		1.287 (0.373–4.441)	0.690		1.258 (0.287–5.519)	0.761

*Abbreviations: SCH* subclinical hypothyroidism; *BMI* body mass index; *FT4* free thyroxine; *TC* total cholesterol; *TG* triglyceride; *LDL-C* low density lipoprotein cholesterol; *HDL-C* high density lipoprotein cholesterol; *GDM* gestational diabetes mellitus; *PROM*, premature rupture of fetal membranes; *FGR* fetal growth restriction; *L-T4* levothyroxine

Crude: No adjustment. Model 1: Adjusted for age and L-T4 use. Model 2: Adjusted for the same variables as Model 1 as well as drinking and smoking. Model 3: Adjusted for the same variables as Model 2 as well as pre-BMI.

^*^P-values < 0.05 was considered statistically significant

### Baseline information for pregnant women with SCH predicts adverse pregnancy outcomes

As depicted in Table 6, among pregnant women with SCH, TSH was predictive capability for preterm birth (AUC = 0.624, 95% CI: 0.535 ~ 0.714, *P* = 0.017) and FGR (AUC = 0.608, 95% CI: 0.546 ~ 0.669, *P* = 0.001), as showed by ROC curve analysis. The established thresholds were 4.565 mIU/L and 5.575 mIU/L, respectively.

**Table 6:** Optimal cut-off points of baseline information in pregnant women with SCH for predicting pregnancy outcomes

Outcomes		AUC (95%CI)	*P*-values		Sensitivity (%)		Specificity (%)		Youden index		Cut-off point
TSH (mIU/L)											
Preterm birth		0.624(0.535, 0.714)	0.017^*^		64.30		35.70		0.232		4.565
FGR		0.608(0.546, 0.669)	0.001^*^		45.20		73.60		0.187		5.575
FT4 (pmol/L)											
Pregnancy loss		0.407(0.325, 0.490)	0.029^*^		95.90		5.00		0.009		12.535
TPOAb(U/mL)											
Gestational hypertension		0.646(0.530, 0.763)	0.026^*^		0.500		0.775		0.275		64.500
FGR		0.602(0.526, 0.678)	0.010^*^		0.700		0.474		0.174		38.650
Preterm birth		0.622(0.503, 0.741)	0.043^*^		0.417		0.819		0.236		97.550
TC (mmol/L)											
Gestational hypertension		0.638(0.550, 0.725)	0.004^*^		73.00		45.00		0.279		4.300
Preeclampsia/Eclampsia		0.610(0.513, 0.707)	0.023^*^		59.50		59.60		0.190		4.365
GDM		0.585(0.535, 0.635)	0.001^*^		48.30		66.50		0.148		4.475
TG (mmol/L)											
Caesarean Section		0.564(0.526, 0.603)	0.001^*^		41.40		70.40		0.119		1.195
Gestational hypertension		0.616(0.516, 0.716)	0.017^*^		59.50		62.50		0.220		1.135
Preeclampsia/Eclampsia		0.629(0.531, 0.726)	0.008^*^		40.50		86.00		0.266		1.605
GDM		0.662(0.617, 0.707)	0.000^*^		88.40		36.90		0.253		0.855
Preterm birth		0.613(0.516, 0.711)	0.029^*^		37.50		83.70		0.212		1.535
Macrosomia		0.613(0.538, 0.687)	0.004^*^		50.80		71.00		0.218		1.255
LDL-C (mmol/L)											
Gestational hypertension		0.637(0.552, 0.722)	0.005^*^		51.40		73.90		0.252		2.575
Preeclampsia/Eclampsia		0.659(0.558, 0.760)	0.001^*^		51.40		81.90		0.332		2.785
GDM		0.593(0.544, 0.643)	0.000^*^		53.70		62.60		0.164		2.335
Age (year)											
Caesarean Section		0.570(0.523, 0.609)	0.000^*^		31.50		79.00		0.105		33.500
Pregnancy loss		0.653(0.579, 0.727)	0.000^*^		73.50		49.60		0.231		30.500
GDM		0.654(0.604, 0.704)	0.000^*^		55.10		71.90		0.270		32.500
Pre-BMI (kg/m^2^)											
Caesarean Section		0.590(0.552, 0.628)	0.000^*^		63.70		51.50		0.152		20.810
Gestational hypertension		0.734(0.655, 0.813)	0.000^*^		88.10		62.40		0.435		22.048
Preeclampsia/Eclampsia		0.632(0.530, 0.733)	0.007^*^		54.10		74.50		0.286		23.530
GDM		0.625(0.576, 0.673)	0.000^*^		66.70		54.20		0.208		21.114
Preterm birth		0.646(0.550, 0.741)	0.005^*^		53.10		76.40		0.295		23.788

*Abbreviations: SCH* subclinical hypothyroidism; *BMI* body mass index; *TSH* thyroid-stimulating hormone; *FT4* free thyroxine; *TC* total cholesterol; *TG* triglyceride; *LDL-C* low density lipoprotein cholesterol; *HDL-C* high density lipoprotein cholesterol; *GDM* gestational diabetes mellitus; *PROM*, premature rupture of fetal membranes; *FGR* fetal growth restriction. ^*^P-values < 0.05 is considered statistically significant

Together with pre-BMI data, the TC, TG, and LDL-C concentrations during pregnancy in women with SCH exhibited predictive value for gestational hypertension, preeclampsia/eclampsia, and GDM (TC: AUCs were 0.638, 0.610, and 0.585, respectively; TG: AUCs were 0.616, 0.629, and 0.662, respectively; LDL-C: AUCs were 0.637, 0.659, and 0.593, respectively; pre-BMI: 0.734, 0.632, and 0.625, respectively).

**Fig. 2:**
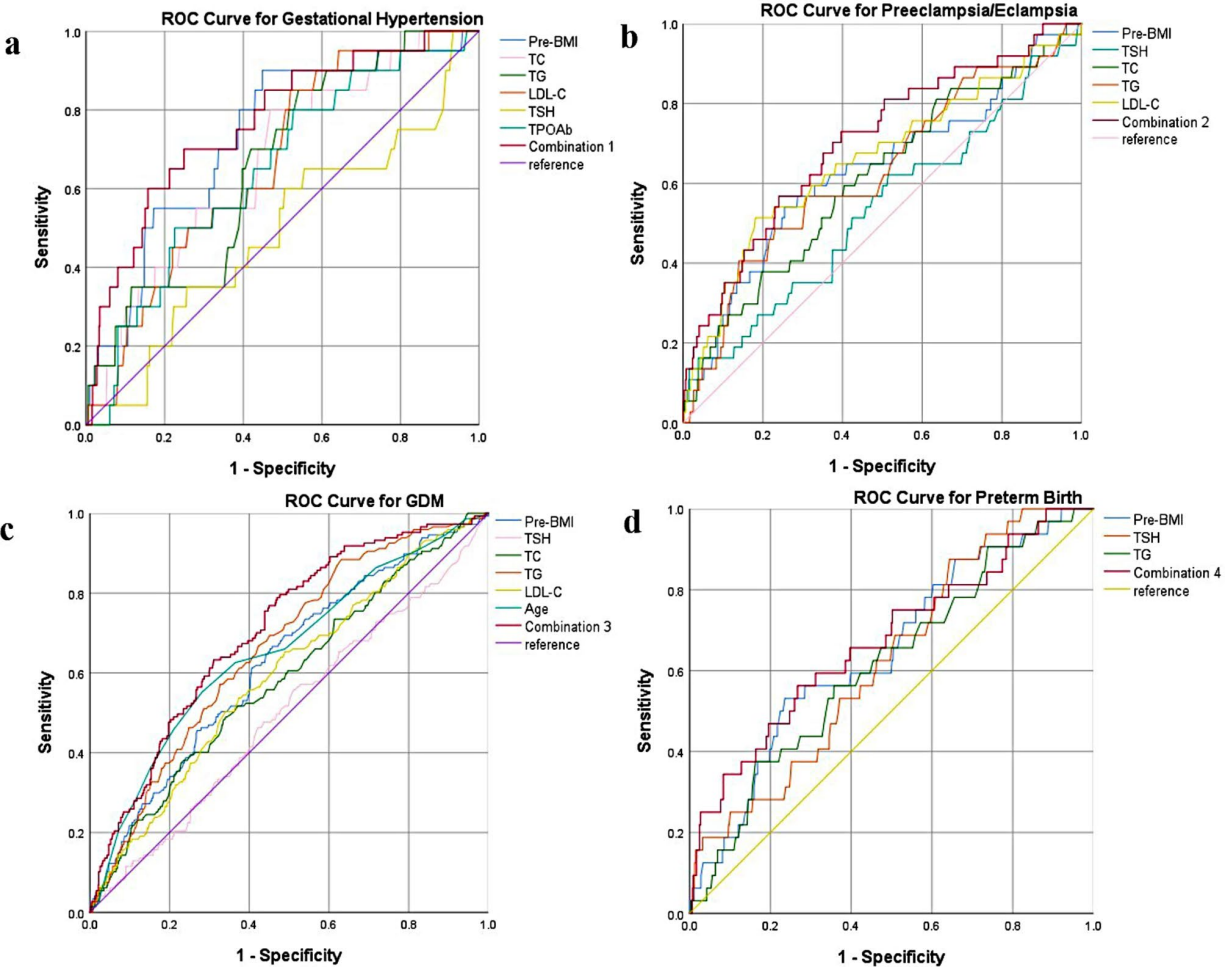
ROC for metabolic markers in the first trimester in predicting adverse outcomes in subclinical hypothyroid. **(a)** ROC curve in predicting gestational hypertension. Combination 1: TC, TG, LDL-C, pre-BMI, TSH and TPOAb. **(b)** ROC curve in predicting preeclampsia/eclampsia. Combination 2: TC, TG, LDL-C, pre-BMI, and TSH. **(c)** ROC curve in predicting GDM. Combination 3: TC, TG, LDL-C, pre-BMI, TSH, and age. **(d)** ROC curve in predicting preterm birth. Combination 4: TG, pre-BMI, and TSH. *Abbreviations: GDM* gestational diabetes mellitus; *TSH*, thyroid-stimulating hormone; *TC*, total cholesterol; *TG*, triglyceride; *LDL-C*, low density lipoprotein cholesterol; *Pre-BM*I, pre-pregnancy body mass index

Furthermore, a combination of factors proved more effective in predicting certain adverse outcomes. Specifically, combining pre-BMI, TC, TG, LDL-C, TSH, and TPOAb demonstrated better predictive power for the occurrence of gestational hypertension (AUC = 0.767 95% CI: 0.661 ~ 0.873, *P* < 0.001, Fig. 2a). Combining pre-BMI, TC, TG, LDL-C, and TSH was suitable for predicting preeclampsia/eclampsia (AUC = 0.704 95% CI: 0. 615 ~ 0.793, *P* < 0.001, Fig. 2b). Additionally, combining TSH, TC, TG, LDL-C, pre-BMI, and age was found to be more suitable for predicting the occurrence of GDM (AUC = 0.706 95% CI: 0.662 ~ 0.749, *P* < 0.001, Fig. 2c), and combining TSH, TG, and pre-BMI was more suitable for predicting the occurrence of preterm birth (AUC = 0.672 95% CI: 0.570 ~ 0.774, *P* = 0.001, Fig. 2d).

## Discussion

This study, which concentrated on women with SCH in their first-trimester pregnancy, indicated that those with dyslipidaemia exhibited elevated incidences of gestational hypertension, preeclampsia/eclampsia, GDM, and low birth weight compared to those without dyslipidaemia. Furthermore, elevated TG dyslipidaemia was correlated with elevated gestational hypertension risk, and elevated LDL-C dyslipidaemia was correlated with elevated preeclampsia/eclampsia risk independent of age, pre-BMI, smoking status, alcohol use, and L-T4 use. Additionally, the combination of lipid and TSH levels had good predictive power for the above pregnancy outcomes.

Dyslipidaemia significantly impacts the long-term health of both mothers and offspring [19, 20]. Elevated TG levels in early pregnancy were shown in previous studies to be correlated with raised risks of gestational hypertension, preeclampsia, GDM, caesarean section, preterm birth, macrosomia, and LGA infants [21, 22]. Additionally, elevated concentrations of LDL-C have been related to an elevated risk of GDM, preterm birth, macrosomia, and intrahepatic cholestasis in pregnancy [23, 24]. Li et al. [25] reported that in women with GDM, TG levels were elevated and HDL-C levels were reduced, and this was combined with higher incidences of caesarean section and macrosomia. Furthermore, within the context of metabolic syndrome, the presence of dyslipidaemia was found to elevate susceptibility to preeclampsia [26]. Notably, this investigation revealed an association between elevated TG and LDL-C dyslipidaemia in early pregnancy and gestational hypertension and preeclampsia/eclampsia in SCH patients.

Among the causes of dyslipidaemia, hypothyroidism is regarded as the most common secondary cause [27]. Thyroid hormones and TSH are significant factors in lipid metabolism disorders [28]. TSH enhances the activation of key enzymes, including sterol regulatory element binding protein 2 and 3-hydroxy-3-methyl-glutaryl coenzyme A reductase, thereby enhancing the accumulation and synthesis of TC and TG within both hepatocytes and adipocytes through distinct signalling pathways [29, 30]. Additionally, an increase in the serum TSH concentrations coincides with the synchronous upregulation of proprotein convertase subtilisin/kexin type 9, causing a reduced LDL-C clearance [31]. A meta-analysis demonstrated an association between elevated values of TG, LDL-C and TC and SCH, whereas the association with HDL-C levels was less clear [32].

Dyslipidaemia constitutes a pivotal risk factor for cardiovascular ailments. The accumulation of lipids, which results in foam cell formation, along with hyperlipidaemic stress-induced inflammation and oxidative stress, are significant precursors to atherosclerotic cardiovascular diseases [33]. The nuclear receptor peroxisome proliferator-activated receptor-γ, which is present in preeclamptic placentas, is stimulated by high levels of TG and free fatty acids, and it ultimately results in abnormal placental vasculature [34]. SCH also frequently coincides with alterations in myocardial function [35], vascular abnormalities [36], and shifts in lipid metabolism [32], potentially heightening the risk of cardiovascular events. Thus, pregnant women with SCH and dyslipidaemia face an elevated risk of developing gestational hypertension and preeclampsia/eclampsia. It is recommended that hypothyroidism is screened for in individuals with dyslipidaemia, particularly those with hypercholesterolemia [37]. Recent studies have suggested that treatment with L-T4 can lead to improvements in TC and LDL-C concentrations in patients with SCH during pregnancy [38]. However, another study reported that using L-T4 in SCH patients to mitigate the risk of cardiovascular disease may not yield unequivocal benefits [39]. In addition, the risk of GDM increases in patients with SCH. TSH can directly reduce the synthesis and secretion of insulin in pancreatic β cells and can also affect insulin resistance [40]. Additionally, thyroid marker levels in early pregnancy are linked to an elevated incidence of GDM. This risk is largely influenced by specific lipid species [41]. Moreover, lipid biomarkers in the first trimester are associated with GDM and have been shown to be strongly predictive of GDM [42].

Notably, pre-BMI has a significant impact on pregnancy outcomes. After accounting for pre-BMI, the association between dyslipidaemia and adverse pregnancy outcomes also changed. Prospective data showed that individuals who were obese (BMI ≥ 25 kg/m^2^) or overweight (23 kg/m^2^ ≤ BMI ≤ 24.9 kg/m^2^) before pregnancy were at increased risk of GDM, preeclampsia, caesarean section, preterm, stillbirth, macrosomia, and LGA but were at decreased risk SGA [43]. Furthermore, a retrospective observational study showed that LGA was independently associated with BMI only in women without GDM [44]. This study demonstrated that pre-BMI independently predicted gestational hypertension during pregnancy in women with SCH, and the ideal cut-off point was 22.048 kg/m^2^. Moreover, age and pre-BMI during pregnancy are both important influencing factors on lipid metabolism. Studies have shown that pre-BMI is the main factor determining the rate of change of TC and LDL-C concentrations [45], and active intervention before or during the first trimester is more effective [46]. In addition, elevated TSH levels have been correlated with an increased BMI [47]. Furthermore, considering lipid levels, thyroid function, age and pre-BMI in pregnant women with SCH had good predictive effects on pregnancy outcomes.

### Study strengths and limitations

As a cohort study during pregnancy, the present study had a large sample size and concentrated on how dyslipidaemia affects unfavourable pregnancy outcomes in women with SCH. Normal reference ranges for lipids in early pregnancy were established in this study and L-T4 use during pregnancy was included as an adjusting variable in the analyses. However, several limitations warrant consideration. First, the research focused on a specific geographical area, potentially introducing bias. Second, thyroid-related hormones and lipids were assessed only in the first trimester, overlooking the dynamic interplay between SCH and lipids throughout pregnancy. Third, thyroid autoimmune-related indicators, such as thyroid peroxidase antibodies and thyroglobulin antibodies, were incorporated into this study, potentially bearing relevance to the mechanistic underpinnings of dyslipidaemia in SCH during pregnancy and its associated adverse outcomes. Consequently, larger sample sizes and well-designed multicentre studies are imperative to corroborate the present findings and to gain a comprehensive understanding of the intricate interplay among dyslipidaemia, thyroid dysfunction, and unfavourable pregnancy outcomes.

## Conclusions

Dyslipidaemia in the first trimester was positively linked to gestational hypertension, preeclampsia/eclampsia, GDM, and low birth weight, which was more affected by pre-BMI. Analysing unfavourable pregnancy outcomes for women with SCH requires a comprehensive assessment of both thyroid function and lipid profiles. In addition, improving the pre-BMI in women with SCH may be beneficial for reducing the risk of adverse pregnancy outcomes.

## Data Availability

The datasets generated during and/or analyzed during the current study are available from the corresponding author on reasonable request.

## Abbreviations

SCH: Subclinical hypothyroidism
TSH: Thyroid stimulating hormone
FT4: Free thyroxine
L-T4: Levothyroxine
TC: Total cholesterol
TG: Triglyceride
HDL-C: High density lipoprotein cholesterol
LDL-C: Low density lipoprotein cholesterol
BMI: Body mass index
CBCS: China Birth Cohort Study
GDM: Gestational diabetes mellitus
PROM: Premature rupture of fetal membranes
FGR: Fetal growth restriction
LGA: Large for gestational age
SGA: Small for gestational age
ROC: Receiver operating characteristic
AUC: Area under the curve
